# Influence of lameness on follicular growth, ovulation, reproductive hormone concentrations and estrus behavior in dairy cows

**DOI:** 10.1016/j.theriogenology.2011.03.019

**Published:** 2011-09-01

**Authors:** M.J. Morris, K. Kaneko, S.L. Walker, D.N. Jones, J.E. Routly, R.F. Smith, H. Dobson

**Affiliations:** a School of Veterinary Science, University of Liverpool, Leahurst Campus, Neston, Wirral CH64 7TE, (UK); b School of Veterinary Medicine, Faculty of Medical Sciences, The University of the West Indies, EWMSC, Mount Hope, Trinidad and Tobago, West Indies; c Department of Veterinary Obstetrics and Gynecology, School of Veterinary Medicine, Azabu University, Tokyo, Japan; d North of England Zoological Society, Chester Zoo, Upton by Chester, CH2 1LH, United Kingdom

**Keywords:** Lameness, Dairy cattle, Follicle, Ovulation, Estrus

## Abstract

The objective of this study was to examine the effect of a chronic stressor, lameness, on reproductive parameters. Seventy cows 30–80 days post-partum were scored for lameness and follicular phases synchronized with GnRH followed seven days later by prostaglandin (PG). Fifteen Lame animals did not respond to GnRH ovarian stimulation. Milk progesterone for 5 days prior to PG was lower in the remaining Lame cows than Healthy herdmates. Fewer Lame cows ovulated (26/37 versus 17/18; P = 0.04) and the interval from PG to ovulation was shorter in Lame cows. In Subset 1 (20 animals), the LH pulse frequency was similar in ovulating animals (Lame and Healthy) but lower in Lame non-ovulators. An LH surge always preceded ovulation but lameness did not affect the interval from PG to LH surge onset or LH surge concentrations. Before the LH surge, estradiol was lower in non-ovulating cows compared to those that ovulated and estradiol concentrations were positively correlated with LH pulse frequency. In Subset 2 (45 cows), Lame ovulating cows had a less intense estrus than Healthy cows, although Lame cows began estrus and stood-to-be-mounted earlier than Healthy cows. In conclusion, we have identified several parameters to explain poor fertility in some chronically stressed animals. From 30 to 80 days post-partum, there was a graded effect that ranged from 29% Lame cows with absence of ovarian activity, whereas another 21% Lame cows failed to express estrus or ovulate a low estrogenic follicle; in 50% cows, many reproductive parameters were unaffected by lameness.

## 1 Introduction

Many studies investigating the mechanisms involved during stress-induced sub fertility have used models of acute or repeated acute stress (so-called chronic) in rodents and ruminants held under experimental conditions [1–5]. However, to be meaningful and relevant to the real world, it is necessary to examine truly chronic spontaneous stressors. Cows that have been continuously lame for several weeks require more inseminations per pregnancy and have a lower pregnancy rate to the first insemination after calving [6–8]. Thus, lameness is an appropriate naturally occurring chronic stressor for study.

The incidence of estrus behaviour is similar in lame cows compared to healthy herd-mates and is accompanied by similar profiles of estradiol in daily milk samples although estrus intensity is lower [9,10]. Thus, lame cows do secrete estradiol, many express estrus and are inseminated but the pregnancy rate to first insemination is ∼10 % lower [6]. Also, it has been suggested that there is a greater incidence of persistent follicles (cysts) in lame cows [11]. This implies a failure of ovulation, and hence the reduced fertility in lame cows might be due to the absence of an adequate ovulatory surge of LH in some cows. Furthermore, studies in sheep have indicated that acute stressors, such as transport or insulin during the follicular phase, lower LH pulse frequency and delay or block the LH surge [12] but the effect of chronic stressors on the patterns of LH (pulses and surge) in ruminants has not yet been investigated.

Our previous work in cattle established lower estrus intensity in lame dairy cows but similar estradiol daily milk profiles [9,10]. This was surprising as the latter hormone is a close regulator of both estrus and pre-ovulatory LH surges in healthy animals [12]. Thus, to further investigate possible mechanisms by which some chronically lame cows are less fertile than healthy herd-mates, we increased the frequency of our observations to test the hypotheses that: (a) the rates of follicular growth are slower; (b) the relationship between plasma estradiol and progesterone profiles is disrupted (and as a consequence estrus expression is disturbed); (c) the frequency of plasma LH pulses is reduced; and (d) the plasma LH surge occurs at the wrong time or is more likely to be totally absent.

## 2 Materials and methods

### 2.1 Animals

This study was performed under a UK Home Office license for work on living animals and with the approval of the University of Liverpool Ethical Review Process. The study was conducted on two commercial farms with 230 and 150 cows from May to November in two successive years. A total of 25 cows were used in Year 1, and 45 cows in Year 2, with similar numbers on each farm in both years. Overall, 70 multiparous lactating Holstein cows were enrolled—healthy cows and those with no confounding clinical conditions except lameness. There was no selection regarding reproductive status, i.e., whether oestrus had been observed. As there were no post-calving or pre-breeding checks being carried out, the ovarian status was unknown at the start of the study. Cows entered the study once between 30 to 80 days post-partum and at any one time, no more than 12 cows were monitored. In the first year, LH profiles were examined in a subset of 2 x 10 cows (5 cows were not frequently sampled); in the second year, all 45 cows were the focus of frequent behavioral observations (methodologies are detailed below). Each herd averaged ∼9000 kg milk per lactation with milking (including study cows) starting at 06.00 h and 16.00 h each day. During the study, animals on one farm were out at grass with a supplementary Total Mixed Ration (TMR) fed indoors for one hour immediately after each milking. Cows on the second farm were kept inside throughout and fed TMR *ad libitum*. Cows had been routinely hoof trimmed at the end of the previous lactation.

The cows were scored for lameness by the same observer throughout using a standardised 1 to 5 system [13]. Lameness scoring was performed weekly from three weeks before estrus cycle synchronization for a total of five weeks to allow the calculation of a mean score for this chronic condition. Animals with a mean score <1.5 were classified as Non Lame and those with ≥1.5 or more were classified as Lame. Animals that were non-lame were defined as Healthy.

It has been suggested that body condition score (BCS) and high somatic cell counts (SCCs) may influence fertility [14,15], so these parameters were also measured for inclusion in statistical analyses. BCS was determined weekly by the same observer throughout using a 1 to 5 (low to high) scoring system [16]. Animals with mean BCS <1.5 were classified as low body condition score (Low BCS) and those with a mean score of ≥1.5 to 3.0 were classified as moderate body condition score (Moderate BCS). Individual cow pooled milk SCCs were measured every four to six weeks by commercial companies employed by the individual farms (National Milk Records PLC, Chippenham, UK or the Cattle Information Service, Watford, UK). Animals with clinical mastitis (presence of clots or watery milk, with or without inflamed teats) were excluded from the study. The SCC of the cows immediately prior to estrous cycle synchronization was used to define the prevailing status of the cow. A cell count <100,000 cells/ml was classified as Low SCC and a count ≥100,000 cells/ml was classified as High [15].

### 2.2 Estrous cycle synchronization

Ovarian follicular phases were synchronized to facilitate frequent ultrasound examinations and blood sampling. As we have previously observed that endogenous progesterone concentrations are lower in the late luteal phase of lame animals [10], exogenous progesterone was avoided in the synchronization protocol. Thus, cows received 100 μg buserelin (GnRH; 2.5 ml Receptal; Intervet, Milton Keynes, UK) at morning milking followed seven days later by 500 μg cloprostenol (PG; 2ml Estrumate; Schering-Plough, Uxbridge, UK).

### 2.3 Milk sampling

Milk samples for hormone analysis were taken every other day for 3 weeks before GnRH administration and then daily until the day after PG injection. Between 2 to 7 days after PG, the frequency of collections increased to twice daily, and then daily for 3 weeks to monitor the subsequent progesterone profile. All samples were taken just before milking and immediately stored at −20 °C without preservative.

### 2.4 Ultrasonography

The ovaries of all animals were scanned at 12h intervals *per rectum* with a Concept/ MCV Veterinary Ultrasound Scanner using a 7.5 MHz linear array probe (Dynamic Imaging, Livingstone, UK) from PG administration until ovulation or the appearance of a new follicular wave. Follicles were identified as non-echogenic structures with a defined border between the follicular wall and antrum. Corpora lutea were identified as grainy echogenic structures with a distinct demarcation from the less echogenic normal ovarian stroma [17]. Diameters were calculated as the average of the largest diameter and a perpendicular measurement. Dominant follicles were defined as those that achieved an internal diameter ≥10 mm in the absence of other actively growing follicles [18,19]. Ovulation was considered to have occurred when a follicle >10 mm was absent at the next ultrasound examination 12 h later; the time of ovulation was taken as 6 h before the sudden disappearance of the largest follicle.

### 2.5 Blood sampling

In year 1, LH pulse and surge secretion patterns were examined in Subset 1 comprising two groups of 10 frequently bled animals on one farm. To facilitate frequent blood sampling, catheters prepared from silastic tubing (0.040 inches ID, 0.086 inches OD, 0.023 inches wall; Sani-Tech, Bio Pure Technology Ltd., Denmead, UK) were placed in the left jugular vein under local anaesthesia just before the PG injection. The external extruding part of the catheter was protected by zinc oxide plaster tape super-glued to the skin. Between samplings, the catheters were filled with heparinised saline (50 i.u./ml) and occluded with a capped leur-lock blunted needle which protruded through the plaster tape. Prophylactic antibiotic (Ceftiofur 1 mg/kg; Excenel RTU; Pfizer Ltd., Sandwich, UK) was administered subcutaneously at catheter insertion and at removal 5 to 6 days later. Blood samples were taken at PG injection and 24 h later; and, to assess pulse parameters, the frequency was increased to every 15 min from 38 to 46 h after PG. Subsequently, sampling continued every 2 h until ovulation was identified by ultrasonography. If ovulation did not occur, 2 h sampling continued until the appearance of a new follicular wave (approximately 5 to 6 days after PG injection). During the eight-hour period of sampling every 15 min, cows were restrained by placing a rope across the back of a free access stall/cubicle in which they could sit or stand with access to food and water. Thereafter, cows were restrained in these stalls for ∼10 min at each blood sampling and then immediately released to have free access to a total area of 200 m^2^.

### 2.6 Hormone assays

Progesterone, was analyzed as ‘pregnane metabolites’ in 50 μl whole milk samples using an established EIA assay [10]. Samples were compared with a series of prepared standard concentrations using progesterone from Sigma-Aldrich, Poole, UK. The minimum detectable amount was 0.015 ng/ml; and the intra- and inter-assay coefficients of variation were 7.8 % and 15 %, respectively.

Estradiol was measured in 0.5 ml plasma after extraction with di-ethyl ether using an MAIA kit manufactured by Biodata S.p.A, Roma, Italy, using a previously described modification [9]. Samples were compared with a standard preparation obtained from Sigma-Aldrich, Poole, UK. The minimum detectable amount was 0.2 pg/ml; the intra- and inter-assay coefficients of variation were 13.5 % and 16.5 %, respectively.

LH was analyzed using 50μl plasma to examine pulses, whereas samples to measure the LH surge were diluted 1:5 with phosphate buffer, pH 7.0. The assay was an EIA modified from [20]. Briefly, the antibody used was LH 518-B7 raised in a mouse against bovine LH [21]. The antibody working dilution was 1:800,000. Bovine LH (NIH-bLH-B10, AFP-5551B) and ovine LH (NIH-oLH-518) for standards and enzyme labeling were obtained from Prof AF Parlow, Torrance, California, USA. Enzyme labeling was performed using an EZ-Link Sulpho- NHS- LC- Biotinylation Kit Product # 21430 from Pierce, Rockford, Illinois, USA. The biotynylated ovine LH label was used at a working dilution of 1:100,000. The minimum detectable amount was 0.15ng/ml, and the intra- and inter-assay coefficients of variation were 3.5 % and 15.5 %, respectively. An LH surge was defined as an increase in LH concentrations >8 ng/ml for two consecutive samples taken 2 h apart [18].

### 2.7 Visual observation of estrus behavior

In year 2, Subset 2 comprising 45 cows was observed for estrus behavior for 7 days following PG on both farms with conditions and methodology as described above. Briefly, in addition to the twice daily scanning and blood sampling during the follicular phase, the frequency and intensity of eight different behavioral signs of estrus were observed for 30 min every 3 h. The signs of estrus were analyzed using a weighted scoring method [22]; cows received ‘points’ based on the number of times a behavioral sign of estrus was observed in a 30 min observation period (Table 1). An animal was considered to be in estrus when the sum of points in a consecutive 30 min observation period exceeded 100 points. Due to the 3 hourly observation regime, the onset of behavior was defined as 1.5 h before the first positive observation and the end as 1.5 h after the last positive observation. The total number of points received over a whole estrus period was considered as a measure of intensity.

### 2.8 Statistical analysis

Statistical differences were considered when P ≤ 0.05. All data were analyzed using Minitab (Version 14; Minitab Inc. Pennsylvania, USA). Follicular growth was determined as the mean daily increase in follicular diameter between time of PG administration and last ultrasound scan before ovulation. Farm, Year, BCS and SCC were included in all statistical models as possible covariates but were found not to be significant and so removed from the final models.

Correlations between variables were examined using Pearson's correlation or Pearson's ranked correlation for non-parametric data. The association between animals that ovulated and lameness category was investigated by χ-square analysis. Behavioral differences between groups were examined with Students *t* test. Further statistical methods included GLM ANOVA using Bonferroni simultaneous comparisons with the fixed factors of lameness and ovulation. For GLM ANOVA repeated measures, lameness and ovulation factors were nested within individual animals. LH pulse frequencies were compared using Mann-Whitney tests for non-parametric data. Assessment of the time intervals from PG to estrus behaviors involved Regression with Life tests (survival analysis).

### 2.9 LH Pulse Analysis

Pulses of LH secretion above baseline were identified using a PC-PULSAR pulse analysis program (Gitzen & Ramirez, University of Illinois, USA. Version 3.0) based on the Pulsar algorithm [23]. G-values used were G1 = 3.8, G2 = 2.6, G3 = 1.9, G4 = 1.5 and G5 = 1.2. Baxter parameters were derived for each assay from which data was used and were specific for each animal [24]. Contentious pulses were re-examined and confirmed by establishing the absence of overlap in errors derived from both G1 x Baxter Standard Deviations (SD) and G1 x real SD of the duplicates. Pulse frequency was then calculated by dividing the number of complete pulses by the duration of the sampling period.

## 3 Results

A ‘non-luteal' progesterone concentration (0.17 ng/ml) was calculated as the mean follicular phase concentration plus twice the SD of 18 Healthy cows [10]. Progesterone profiles revealed 15 Lame animals that did not respond to estrus synchronization (progesterone values remained at or below 0.17 ng/ml until Day 4 after PG, and none ovulated); these were categorized as Lame Non Responders. The remaining animals that had an increase in progesterone in response to synchronization were categorized into those that did or did not ovulate, to form the following groups that reflect increasing disruption by lameness: non lame ovulated (Healthy, n = 17); non lame non ovulated (n = 1; this cow was removed from all further analyses, except the ovulation rate data in the following paragraph); Lame Ovulated (n = 26); and Lame Non Ovulated (n = 11). Hence, along with the 15 Lame Non Responders, a total of 69 animals were studied further.

Overall, a greater proportion of Healthy (17/18) than Lame (26/52) cows ovulated (P *=* 0.001; χ -square = 11.15). When the 15 Lame Non Responder cows were removed from the analysis, fewer Lame cows still had not ovulated (P *=* 0.04; χ -square = 4.15).

### 3.1 Follicles

**Fig. 1:**
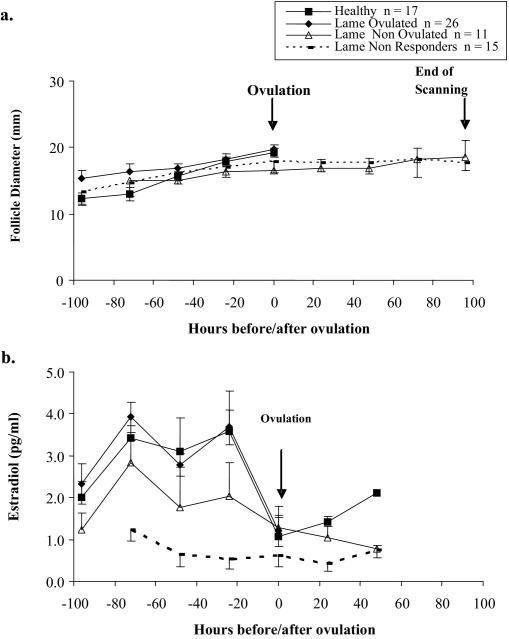
(a) Mean follicular diameter (error bars: SEM), and (b) mean plasma estradiol concentrations (error bars: SEM) in cows of differing lameness status during synchronized follicular phases (GnRH followed seven days later by PG). Data for non-ovulating groups have been aligned to Day 4.5 after PG (closest mean day of ovulation in ovulating cows). Data were collected twice a day but results are shown daily for clarity.

At the time of PG injection, each cow, regardless of group, had an actively growing follicle of at least 10 mm diameter that grew to a maximum of between 14 and 31 mm. The mean follicular growth rate from PG to ovulation was not different between Lame and Healthy cows, whether they ovulated or not (Figure 1a; 1.4 ± 0.2 vs 2.1 ± 0.2 mm/day*;* P = 0.06; n = 69). Maximum diameter of the dominant follicle was defined as the diameter just before ovulation (mean time for Healthy cows = Day 4.4 ± 0.3 after PG), or the diameter on Day 4.5 in those animals that failed to ovulate. The maximum diameters of dominant follicles were not different between Healthy and Lame Ovulated cows (P = 1.0). The maximum diameters of dominant follicles that did or did not ovulate were not different (P = 0.75). For those cows that did ovulate, the time from PG to ovulation was shorter in Lame cows compared to Healthy cows (4.1 ± 0.1 vs 4.4 ± 0.2 days; P = 0.04).

### 3.2 Hormonal analyses in all animals

**Fig. 2:**
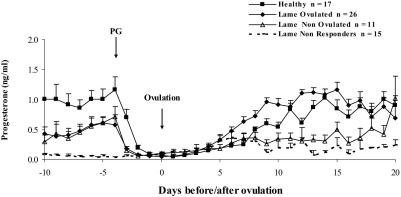
Mean (error bars: SEM) concentrations of progesterone in milk (ng/ml) in cows of differing lameness status prior to, during and following hormonal synchronization (GnRH followed seven days later by PG). Data for non-ovulating groups were aligned to Day 4.5 after PG (closest mean day of ovulation in ovulating cows).

For the period five days prior to PG injection, mean milk progesterone concentrations in Healthy animals were higher than in all three groups of Lame cows (Table 2; Figure 2; P = 0.04). Progesterone concentrations were intermediate in both Lame Ovulated and Lame Non Ovulated cows; and Lame Non Responder animals had the lowest concentrations (Table 2; Figure 2; P = 0.009). Mean milk progesterone concentrations were not different between all groups in the peri-ovulatory period (Day −1 pre-ovulation to Day +1 post ovulation) and on Day 5 (Table 2; Fig. 2). The concentrations on Day 7 were higher in ovulating animals (Healthy and Lame Ovulated groups) than in Lame Non Responder cows; Lame Ovulated animals also had higher concentrations than Lame Non Ovulated animals (Table 2; Fig. 2; P = 0.05). During the mid luteal phase 12–17 d after ovulation, mean progesterone concentrations were higher in Lame Ovulated cows than in Lame Non Ovulated and Lame Non Responder animals (Table 2; Fig. 2; P *=* 0.0003).

Mean plasma estradiol concentrations in samples collected during the period 36 h before ovulation were greater in both groups in which cows ovulated (Healthy and Lame Ovulated; Fig. 1b; P = 0.03) than in the Lame Non Responder group. Values for the Lame Non Ovulated group were intermediate.

### 3.3 Hormonal analyses in frequently blood sampled animals

Subset 1 consisted of 4 Healthy and 16 Lame cows, and 17 out of all 20 responded to estrous cycle synchronization (thus, there were three Lame Non Responders). Twelve of the 17 responding animals ovulated, comprising 4 Healthy and 8 Lame Ovulated cows; leaving 5 Lame Non Ovulated cows (Table 3). All the frequently blood sampled animals had a BCS of 2.5.

**Fig. 3:**
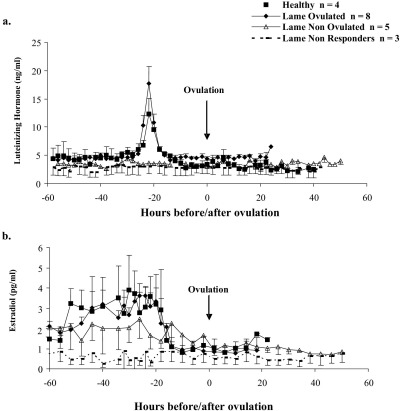
Mean (error bars: SEM) plasma concentrations of a) LH (ng/ml) and b) estradiol (pg/ml) in cows of differing lameness status during synchronized follicular phases (GnRH followed 7 days later by PG). Data for non-ovulating groups were aligned to Day 4.5 after PG (closest mean day of ovulation in ovulating groups).

All the 12 cows that ovulated had an LH surge occurring 72 ± 10 h after PG and 22.2 ± 1.5 h prior to ovulation. These intervals were not influenced by lameness (Healthy vs Lame Ovulated, P *=* 1.0 and 0.2, respectively). Maximum LH (surge) concentrations ranged from 7.3 to 28.8 ng/ml in the ovulating animals but again there was no association with lameness (Table 3; P *=* 0.9). The 5 Lame Non Ovulated and 3 Lame Non Responder cows had no discernible LH surge (range 4.3–6.2 ng/ml) and did not ovulate (Fig. 3a).

During the period 22 to 40 h before ovulation (i.e., during the 18 h period before the LH surge), mean plasma estradiol concentrations in the Lame Non Responder group were lower than in Lame Ovulated and Healthy cows (Table 3; Fig. 3b; P *=* 0.03 and P *=* 0.05, respectively). Over the same period, estradiol concentrations were higher in all the animals that subsequently ovulated (Healthy + Lame Ovulated) than in all non-ovulating cows combined (Lame Non Ovulated + Lame Non Responders; 3.7 ± 0.4 vs 1.4 ± 0.6 pg/ml, P *=* 0.006).

**Fig. 4:**
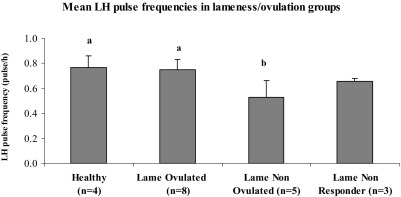
Histogram of mean LH pulse frequency (pulses/h + SEM) for all animals in each group (values ^a^*versus*^b^ P *=* 0.04).

Mean LH pulse amplitude (ng/ml) was not different between all groups (n = 20, P *=* 0.6; Healthy: 0.89 ± 0.17; Lame Non Ovulated: 0.68 ± 0.17; Lame Ovulated: 1.3 ± 0.23; Lame Non Responder: 0.53 ± 0.16). The mean LH pulse frequency was not different between Healthy and Lame Ovulated animals (P *=* 0.9) but Lame Non Ovulated animals had lower frequencies (Fig. 4; P = 0.04). Lame Non Responder cows had a similar LH pulse frequency as Lame Ovulated animals (Fig. 4; P = 0.06). LH pulse frequency was lower in all animals that subsequently did not ovulate (Lame Non Ovulated + Lame Non Responders) than in all ovulating cows combined (Healthy + Lame Ovulated) (P *=* 0.001). LH pulse frequency was positively correlated with mean estradiol concentrations both at the time of pulse sampling (r = 0.59, P *=* 0.006), and over the period 18 h before the LH peak (r = 0.64; P *=* 0.002).

### 3.4 Visual observations of estrus behavior

Subset 2 cows were observed for estrus behavior and comprised 13 Healthy animals; 15 Lame Ovulated; 6 Lame Non Ovulated; and 11 Lame Non Responders. Only three of the Lame Non Ovulated or Lame Non Responder cows showed any behavioral sign (and then only 1 or 2 sniffs each throughout the whole period observed) and hence none of the 17 animals in these two groups were considered to have been in estrus (minimum score requires >100 points in a 30 min period). Out of the initial 45 cows, 28 (62%) displayed signs of estrus, in which there were fewer Lame cows (13/13 Healthy vs 15/32 Lame; P *=* 0.001; χ-square = 11.1). When the 11 Lame Non Responder animals were removed from the statistical analysis, there were 28/34 cows (82%) observed in estrus, but still, there were fewer Lame cows in estrus (13/13 Healthy vs 15/21 Lame; P *=* 0.03; χ-square = 4.5).

Lame Ovulating cows had a less intense estrus and a lower maximum estrus score in any 30 min period than the Healthy cows (Table 4; P = 0.02 and 0.05, respectively). However, the intervals from PG to start of estrus and to the first stand-to-be-mounted (STBM) were shorter for the Lame cows than Healthy cows (Table 4; P = 0.004 and 0.002, respectively). All the animals that displayed estrus ovulated and vice versa, irrespective of lameness status.

## 4 Discussion

Four distinct groups of cows in the study were revealed when categorized by lameness and reproductive activity, namely: 1) Lame cows that were totally unresponsive to the synchronization regime—29% of all Lame cows; 2) Lame cows that responded to synchronization but did not ovulate—21% of all Lame cows; 3) Lame cows that responded to synchronization and ovulated—50% of all Lame cows; and 4) Healthy animals that responded to estrus synchronization, all of which ovulated within the study period. Thus, in the period 30–80 days post-partum, there was a graded effect of lameness in dairy cows, ranging from total shut-down of ovarian activity (not even responding to exogenous hormonal stimulation) to ovarian activity being unaffected by lameness.

Others have suggested that some lame cows have a delayed return to regular ovarian/ovulation cyclicity in the early postpartum period [25]. The present study advances those observations, by showing that 50% of Lame cows did not ovulate or express estrus even after external hormonal stimulation in the postpartum period from 30–80 days after calving. We propose that poor responses to endogenous or exogenous hormones contribute to the lower fertility in some lame cows after insemination at this economically crucial time [6,8].

All animals in the present study had a follicle greater than 10 mm that subsequently grew in all groups indicating that mechanisms controlling follicular emergence, selection and physical growth were unaffected by lameness, regardless of prior progesterone status. These mechanisms appear, therefore, to be different from those during heat stress in which growth and size were affected [26,27]. Furthermore, the maximum diameters achieved were similar in both Healthy and Lame cows—although, in Non Ovulating Lame cows, the structure persisted for longer. Furthermore, we have shown for the first time that the large follicles that did not ovulate remained physically present but were not functional, i.e., hormone concentrations in peripheral plasma were basal and another wave of follicles eventually developed. These new observations are consistent with the positive association between lameness and incidence of ovarian cyst diagnosis [11]. Interestingly in the present study, Lame cows ovulated earlier in estrus than Healthy animals; the reason for which is unclear. It is known that the scanning process itself does not influence the time of ovulation [28].

In the early postpartum period of healthy cows, FSH provokes initial follicle growth; adequate LH pulses then stimulate estradiol production by late follicular phase dominant follicles and this is followed sequentially by estrus behavior, an LH surge, ovulation and formation of a good corpus luteum (defined as one producing maximum progesterone concentration for 12–15 d). It requires a fully functional dominant follicle to form a good corpus luteum with a ‘mature’ progesterone profile [29]. Often, the first postpartum CL is ‘immature’ and produces lower maximum concentrations of progesterone for a shorter period (7–9 d; [30,31]). From the new observations in the present study, we suggest that lameness (mediated by an associated low LH pulse frequency) either tends to delay the formation of a good follicle, and hence may delay optimum corpus luteum growth and function [25]; or, despite the emergence of a good follicle, subsequent poor function eventually leads to low progesterone production. Evidence for the latter was provided by our previous study that revealed low progesterone profiles in non-synchronized Lame cows at a similar time after calving [10]. The presence of an immature corpus luteum results in reduced progesterone priming at a critical time for the attainment of hypothalamic responsiveness to estradiol [32]. Whatever the cause, progesterone concentrations in all groups of Lame cows in the present study were lower than in Healthy animals. Furthermore, follicles that had been luteinized by the GnRH synchronization treatment were unable to produce a fully functional corpus luteum before prostaglandin treatment in Lame cows. Our earlier observations on non-synchronized animals have, consequently, been extended by showing that follicles in some lame animals are functionally immature and do not respond to GnRH, although the follicles appear to be of sufficient size.

Prior exposure to progesterone in a luteal phase is undoubtedly important to establish normal estrus behavior and ovulation but the Lame Ovulating and Non Ovulating groups had similar prior progesterone values, although lower than in the Healthy cows. In the ewe, adequate prior progesterone exposure leads to an increase in the density of estradiol receptors in the ventro-medial hypothalamus, thus promoting a further increase in LH pulse frequency, estradiol induction of estrus behavior and an LH surge [33].

However, later in the follicular phase, progesterone concentrations were similar in Healthy and Lame animals, therefore, suprabasal concentrations do not seem to contribute to lameness-induced lowered fertility as suggested in other infertility studies, albeit in non-lame heifers [34]. Furthermore, those animals in the present study that did ovulate (100% Healthy cows and 50% of all Lame cows) had similar progesterone profiles in the following luteal phase. This indicates that the low pregnancy rate in Lame cows is due to failure of ovulation, not failure of luteal support during the early luteal phase previously high-lighted as critical to establish a successful pregnancy [35]. Half the Lame cows had deficient luteal phases, reflecting the absence of ovulation of the synchronized dominant follicle. Noteworthy, however, is the presence of increased progesterone concentrations in 3/11 of the Non Ovulating Lame animals. These were possibly due to spontaneous luteinization of the dominant follicle or ovulation after ultrasonography had stopped.

In a previous study, measuring estradiol in daily milk samples [10] and in the present study when estradiol concentrations were monitored twice daily in plasma, we were unable to detect differences between estradiol profiles in Healthy and Lame cows. This was probably because the measurements were too infrequent during a follicular phase that only lasts 2–3 d. However, estradiol concentrations were lower in plasma samples taken more frequently (every 2 h) from Lame Non Ovulating and Lame Non Responder cows, although the follicles were of similar size in all animals. The presence of a large follicular structure was associated with high estradiol production in those animals with a higher LH pulse frequency; and only those same animals displayed full estrus behavior, had an LH surge and ovulated. It is not yet clear why 50% of the Lame cows had adequate LH pulses and consequently high estradiol concentrations while 21 % Lame cows did not (excluding the 29 % Lame Non responders that had low LH pulses and low estradiol). Once an LH surge occurred, ovulation followed, with no differences in timing or surge concentrations of LH between the Lame and Healthy groups. This adds weight to the suggestion that ovulation occurred once the thresholds of LH pulsatility and of estradiol required for the production of an LH surge were achieved. Estradiol concentrations were higher in the Healthy animals and such animals displayed more intense estrus behavior in agreement with other work [28,36].

The present study is the first to show that spontaneous chronically stressed animals that do not ovulate (lame cows with a BCS of 2.5 in this case) have a low LH pulsatility. Furthermore, both LH pulse frequencies and estradiol concentrations were lower in frequently bled Lame animals that did not display estrus or ovulate than in those animals that expressed estrus and ovulated. Thus, we propose that the chronic stress of lameness in postpartum cows is associated with a graded reduction of ovarian hormone production (ranging from minor to severe as identified above). These effects originate mainly at hypothalamic level because LH pulse frequency is dictated by frequency of GnRH pulses generated from this area of the brain in ruminants [37]. The lower LH pulse frequency in Lame cows that do not ovulate reduces the ability of selected follicles to produce sufficient estradiol to induce intense estrus behavior, an LH surge and ovulation. These new results obtained during chronic stress (albeit in a small number of animals) concur with our previous observations in sheep showing that acute stressors such as transport or sudden hypoglycemia reduce LH pulse frequency that ultimately lower estradiol concentrations [38]. Similarly, in cattle, acute administration of ACTH or transport reduces LH pulse frequency and estradiol resulting in delayed ovulation [18,39]. Concentrations of cortisol are elevated after exposure to these acute stressors but not in chronically lame animals indicating that this particular hormone in the stress axis is not involved [10,40].

Reduced intensity of estrus due to lameness has been previously described [10] and this may also be partially explained by the decrease in progesterone priming which reduces responsiveness to estradiol, thus reducing the intensity of estrus expression [41]. The physical pain associated with lameness may also decreased walking activity and mounting [10]. The variability in the interval between onset of estrus and ovulation reported by others [42] may be a result of the effect of lameness as revealed by the present study. At a practical level, the quicker onset to start of estrus after PG and the earlier start of STBM in relation to ovulation in lame animals may lead to incorrect times of artificial insemination subsequently leading to poor fertility.

In conclusion, the present study has provided evidence that 21 % of animals with a spontaneously occurring stressful condition (lameness) fail to express estrus or ovulate what was an otherwise seemingly normal follicular structure. A further 29 % of Lame animals were incapable of producing a functional follicle in response to exogenous hormonal stimulation. The failure to express estrus and ovulate was undoubtedly associated with a reduced LH pulse frequency, lower estradiol concentrations or responsiveness to estradiol, and the absence of an LH surge. Body condition scores were included in all statistical analyses and no influences were detected on any outcome.

Further work is required to determine why 50 % Lame animals that did have similarly low prior progesterone concentrations did manage to maintain normal LH and estradiol profiles, and ovulate, eventually producing a normal-functioning corpus luteum.

## Figures and Tables

**Table 1:** Behavior scoring scheme: each recorded observation of an estrus sign was scored according to weightings previously described [22].

Behavior	Points
Restlessness	2
Flehmen	3
Vulval sniffing	10
Mounted but did not stand	10
Chin resting	15
Mounting the rear of another cow	35
Mounting the head of another cow	45
Standing to be mounted (STBM)	100

**Table 2:** Milk progesterone concentrations (mean ± SEM; ng/ml) in groups of Healthy or Lame cows.

	Healthy (n = 17)	Lame Ovulated (n = 26)	Lame Non Ovulated (n = 11)	⁎Lame Non Responder (n = 15)
5 days before PG	a0.98 ± 0.14	b0.53 ± 0.07	b0.52 ± 0.12	c0.05 ± 0.01
Day -1 to +1 ovulation	0.06 ± 0.04	0.05 ± 0.01	0.10 ± 0.02	0.30 ± 0.10
Day 5 after ovulation	0.32 ± 0.05	0.37 ± 0.03	0.23 ± 0.06	0.19 ± 0.06
Day 7 after ovulation	a0.59 ± 0.06	a0.60 ± 0.04	b0.33 ± 0.08	b0.29 ± 0.10
Days 12–17 after ovulation	a0.77 ± 0.08	a1.03 ± 0.10	b0.33 ± 0.08	b0.28 ± 0.12

Within rows, values with different superscripts (

a b c ) are different (P = 0.04).

⁎ Lame Non Responder, lack of response to synchronization (see text for definition).

**Table 3:** Mean ± SEM concentrations of estradiol (E2; pg/ml), maximum LH surge values (ng/ml) and incidence of LH surge and ovulation in groups of Healthy and Lame cows that were blood sampled at least every two hours.

	Healthy (n = 4)	Lame Ovulated (n = 8)	Lame Non Ovulated (n = 5)	⁎Lame Non Responder (n = 3)
Mean plasma E2 (pg/ml) during 22–40 h before estimated time of ovulation	a3.6 ± 1.0	c3.7 ± 0.4	2.0 ± 0.8	b^,^^d^0.4 ± 0.2
LH surge observed	4	8	0	0
Maximum (surge) plasma LH values (ng/ml)	c16.5 ± 4.3	c14.6 ± 2.2	d5.2 ± 0.3	d5.2 ± 0.7
Ovulated	4	8	0	0

Within rows,

a versus

b P*=* 0.05;

c versus

d P *=* 0.03.

⁎ Lame Non Responder, lack of response to synchronization (see text for definition).

**Table 4:** Parameters of estrus behavior (mean ± SEM) in Healthy and Lame cows in which behavior was monitored for 30 min every 3 h after prostaglandin injection (PG). STBM, standing to be mounted.

	Healthy (n = 13)	Lame Ovulated (n = 15)
Total oestrus intensity points	a2254 ± 311	b1368 ± 219
Maximum points per 30 min period	a855 ± 100	b600 ± 77
Interval PG to start of oestrus (h)	a80.5 ± 5.0	b70.6 ± 2.8
Interval PG to first STBM (h)	a85.2 ± 5.0	b72.7 ± 3.4

Within rows,

a versus

b P *=* 0.05.
